# Increased copy number at 3p14 in breast cancer

**DOI:** 10.1186/bcr1279

**Published:** 2005-07-06

**Authors:** Ingrid Ljuslinder, Beatrice Malmer, Irina Golovleva, Marcus Thomasson, Kjell Grankvist, Thomas Höckenström, Stefan Emdin, Yvonne Jonsson, Håkan Hedman, Roger Henriksson

**Affiliations:** 1 Department of Radiation Sciences, Oncology, Umeå University, Umeå, Sweden; 2 Department of Medical Biosciences, Medical and Clinical Genetics, Umeå, Sweden; 3 Department of Medical Biosciences, Clinical Chemistry, Umeå University, Umeå, Sweden; 4 Department of Pathology, Umeå University, Umeå, Sweden; 5 Department of Surgical and Perioperative Sciences, Division of Surgery, Umeå University, Sweden

## Abstract

**Introduction:**

The present study was conducted to investigate if chromosome band 3p14 is of any pathogenic significance in the malignant process of breast cancer. Genetic studies have implicated a tumour suppressor gene on chromosome arm 3p and we have proposed *LRIG1 *at 3p14 as a candidate tumour suppressor. The *LRIG1 *gene encodes an integral membrane protein that counteracts signalling by receptor tyrosine kinases belonging to the ERBB family. LRIG1 mRNA and protein are expressed in many tissues, including breast tissue.

**Methods:**

In the present report we analysed the *LRIG1 *gene by fluorescence *in situ *hybridisation (FISH), *LRIG1 *mRNA by quantitative RT-PCR, and LRIG1 protein by western blot analysis. Two tumour series were analysed; one series consisted of 19 tumour samples collected between 1987 and 1995 and the other series consisted of 9 tumour samples with corresponding non-neoplastic breast tissues collected consecutively.

**Results:**

The *LRIG1 *gene showed increased copy number in 11 out of 28 tumours (39%) and only one tumour showed a deletion at this locus. Increased *LRIG1 *copy number was associated with increased levels of *LRIG1 *mRNA (two of three tumours) and protein (four of four tumours) in the tumours compared to matched non-neoplastic breast tissue, as assessed by RT-PCR and western blot analysis.

**Conclusion:**

The molecular function of LRIG1 as a negative regulator of ERBB receptors questions the biological significance of increased *LRIG1 *copy number in breast cancer. We propose that a common, but hitherto unrecognised, breast cancer linked gene is located within an amplicon containing the *LRIG1 *locus at 3p14.3.

## Introduction

Breast cancer is a major cause of death among women. In order to provide optimal treatment, prognostic factors, such as lymph node status and steroid receptor expression, are widely used. In recent years, genetic approaches studying chromosomal aberrations have been suggested as a tool in the process to individualise the adjuvant treatment given to patients. Several studies have been published during the past years with a focus on identifying the genes that contribute to initiation and clinical progression of breast cancer [1,2].

Identification of a germline mutation of *BRCA1 *at 17q21 [3] and *BRCA2 *at 13q12-13 [4] has been an important finding in studies of hereditary breast cancer. Epidermal growth factor receptor (EGFR/ERBB1) and ERBB2 (also known as HER2) overexpression [5-7], p53 inactivation [8,9] and nm23 overexpression [10] also seem to be of clinical prognostic importance. Chromosomal amplifications have been described in breast cancer for several genes, including *MYC *at 8q24 and *ERBB2 *at 17q11.2 [11,12]. Other amplified chromosomal areas detected in breast cancer are 13q31, 17q22-24, 1q41-44 and 20q13. In general, gene amplifications are considered late events in cancerogenesis, even though much is still unknown about the importance of amplifications of specific genes. In breast cancer, amplification of *ERBB2 *correlates with a worse prognosis [13] and amplification of *C-MYC *is associated with progression from carcinoma *in situ *to invasive breast cancer [14]. Cytogenetic analyses of tumours have shown that chromosome 1 is the most frequently altered chromosome in breast cancer [15]. In other breast cancer studies, loss of heterozygosity (LOH) at 3p was the most common chromosomal aberration [16-18]. In a study by Maitra *et al*. [19], LOH in the 3p area was apparent in 87% of breast tumours, and LOH at 3p14.3 in 41% of the tumours. The short arm of chromosome 3 thus likely harbours at least one tumour suppressor gene [20]. The *FHIT *gene localized to 3p14.2, which frequently shows LOH, is also suggested to be a prognostic factor in breast cancer [21,22].

Recently, the human gene *LRIG1 *(leucine-rich and immunoglobulin-like domains 1) was described and localised to chromosome 3p14.3 [23,24]. The *LRIG1 *gene encodes a protein with extracellular leucine-rich repeats and immunoglobulin-like domains, a transmembrane part, and a cytoplasmic tail. LRIG1 acts as a negative regulator of ERBB1-4 by enhancing receptor ubiquitylation and degradation [25,26]. The mechanism involves the recruitment of c-Cbl, an E3 ubiquitin ligase that simultaneously ubiquitylates EGFR and LRIG1 and sorts them for degradation [25].

The role of LRIG1 as a part of a group of proteins that help desensitize receptor tyrosine kinase (RTK) signalling makes it important to study the expression and role of LRIG1 in tumours in which the ERBB receptors have clinical relevance.

The present study was conducted to investigate if the *LRIG1 *gene, mRNA, or protein was deleted or dysregulated in human breast cancer. The *LRIG1 *locus was analysed by fluorescence *in situ *hybridisation (FISH), mRNA was quantified by real-time RT-PCR and protein was analysed by western blot analysis. To further explore how LRIG1 expression was related to growth factor receptor expressions, quantitative RT-PCR of EGFR and ERBB2 was performed. We report an unexpected increase in copy number of the *LRIG1 *locus in 39% of the breast tumours, implicating a breast cancer gene at, or close to, 3p14.3.

## Materials and methods

### Patients and sample preparation

Previously collected (1986 to 1995) samples from 19 patients were included in a first examination (group A). Tumour samples and non-neoplastic breast tissue were then collected from nine patients with breast carcinoma (group B). Clinical characteristics of the patients are presented in Table 1. The study was approved by the local ethics committee. None of the patients had received any treatment prior to specimen collection. In group B, samples of the tumour and a piece of the non-neoplastic breast tissue were collected immediately after excision, one part of each frozen in liquid nitrogen and stored at -80°C, and another part stored in RNA*later *(Ambion inc, Austin, Texas, USA). The other adjacent parts of the tissue samples were fixed in formalin, paraffin embedded and used for routine morphological examination and tumour grading (according to Page *et al*. [27]), immunohistochemical staining and tumour tissue array construction. The preparation of RNA was performed as previously described [23].

### FISH

Freshly frozen breast cancer tissues were disintegrated in methanol:acetic acid solution (3:1; Carnoy's solution) on ice. The nuclei were collected by passing the disintegrated tissues through a nylon mesh (pore size 70 μm) and then centrifuged. Cells were washed in methanol:acetic acid solution (3:1) two to three times at room temperature. FISH slides were prepared by dropping the cell suspension onto glass slides. After air-drying, FISH-slides were immediately used or stored at -20°C. Before hybridisation, FISH-slides were incubated in 75 mM KCl for 20 minutes at 37°C and fixed in Carnoy's solution for 5 minutes at room temperature. After fixation, FISH slides were treated with RNAase (100 μg/ml) for 1 h, followed by washing in 2 × SSC (saline sodium citrate) three times for 2 minutes each time. Finally, the slides were incubated in solution containing 100 μg/ml pepsin in 10 mM HCl for 10 minutes, followed by incubation in PBS for 5 minutes at room temperature and stepwise dehydration in alcohol (70%, 80%, 95%). The BAC clone 751k5 (Invitrogene, Carlsbad, USA), containing the *LRIG1*, was used as the FISH probe. DNA was labelled by nick translation using Spectrum Orange according to the manufacturer's protocol (Abbot Diagnostics, Wiesbaden-Delkenheim, Germany). Probe (10 μl) containing 100 ng DNA, 5 μl Cot-1 DNA in 60% formamide was pre-incubated for 1 h at 37°C and then applied to each slide. Probe and target DNA were denatured simultaneously for 3 minutes at 72°C. Slides were hybridised overnight at 37°C in a humidity chamber. Post-hybridisation washing was performed in 2 × SSC containing 0.3% NP-40. Nuclear counterstaining was done with DAPI solution for 2 minutes. As control, a centromere probe for chromosome 3 was included in the hybridisation solution.

In each case, *LRIG1 *and CEP3 signals were counted in 100 to 200 nuclei by two independent investigators. The presence of at least three signals in more than 20% of the nuclei was the criteria for scoring an increased copy number of *LRIG1*. Analysis was performed using an Axioplan 2 microscope (Carl Zeiss Vision, Hallbergmoos, Germany.) Digital images were captured and stored using Cytovision software (Applied Imaging Corporation, San Jose, USA).

### Cell lines

The breast cancer cell lines MDA-MB-231, MDA-MB-415 and HS 578T were obtained from American type culture collection (Manassas, VA, USA) and ZR-75-1 was kindly provided by Dr J Bergh (Uppsala University, Sweden). The breast cancer cell lines were cultivated in Dulbecco's modified Eagles medium, supplemented with 10% w/v fetal bovine serum and 50 μg/ml gentamicin from Invitrogen AB (Täby, Sweden). The immortalised mammary epithelial cell line hTERT-HME1 was obtained from BD Bioscencies Clontech (Stockholm, Sweden) and cultivated according to the manufacurer's instructions by using media and supplements from Clonetics, Bio Whittaker (Walkersville, MD, USA).

### Quantitative RNA analysis

RNA was prepared from tissue samples by using RNAqueous kit (Ambion inc, Austin, Texas, USA), according to the manufacturer's instructions. Real-time quantitative RT-PCR was performed as previously described [28].

### Western blot analysis

Cell lysates, protein concentrates and immunoprecipitated material were incubated in LDS (lithium dodecyl sulfate) sample buffer for 10 minutes at 70°C followed by electrophoresis on 3% to 8% TRIS-acetate NuPAGE gradient gel. The proteins were thereafter transferred to polyvinylidene difluoride membranes by using an Xcell II Mini-Gel blot module. Gel apparatus, gels, buffers, blotting module, and membranes were from Invitrogen. Non-specific binding was blocked by using incubation of the membranes with 5% w/v non-fat milk powder in TBS containing 0.1% w/v Tween-20. The membranes were thereafter incubated with the primary antibodies at 1 μg/ml followed by peroxidase-conjugated secondary antibodies (Amersham Pharmacia Biotech, Amersham Biosciences, New Jersey, USA). The primary antibodies used were LRIG1-151 [24] and rabbit anti-actin (Sigma-Aldrich St. Louis, Missouri, USA). Visualization was performed by using the enhanced chemiluminescense system ECL-plus, (ECL-advanced and hyperfilm ECL Blotting Detection system kit, Amersham Biosciences, New Jersey, USA). The samples were diluted stepwise by approximately 50% in 3 to 4 steps. The results were analysed visually by three separate investigators and an apparent change between tumour and non-neoplastic tissue of at least 50% was considered convincing.

## Results

### FISH analysis of archived breast cancer samples

To evaluate the number of *LRIG1 *gene copies, FISH was performed on cell nuclei from the archived breast cancer samples (group A). An increased copy number of *LRIG1 *was seen in more than 20% of the nuclei in 7 of the 19 tumours (in most cells three to five signals). The fraction of tumour cells with increased copy number varied between 23% and 79%. Normal signal pattern corresponding to two copies per nucleus was detected in 11 of the 19 tumours, and 1 tumour demonstrated decreased copy number of *LRIG1 *(Table 2).

### FISH Analysis of Fresh Tumour Samples and Breast Cell lines

**Figure 1:**
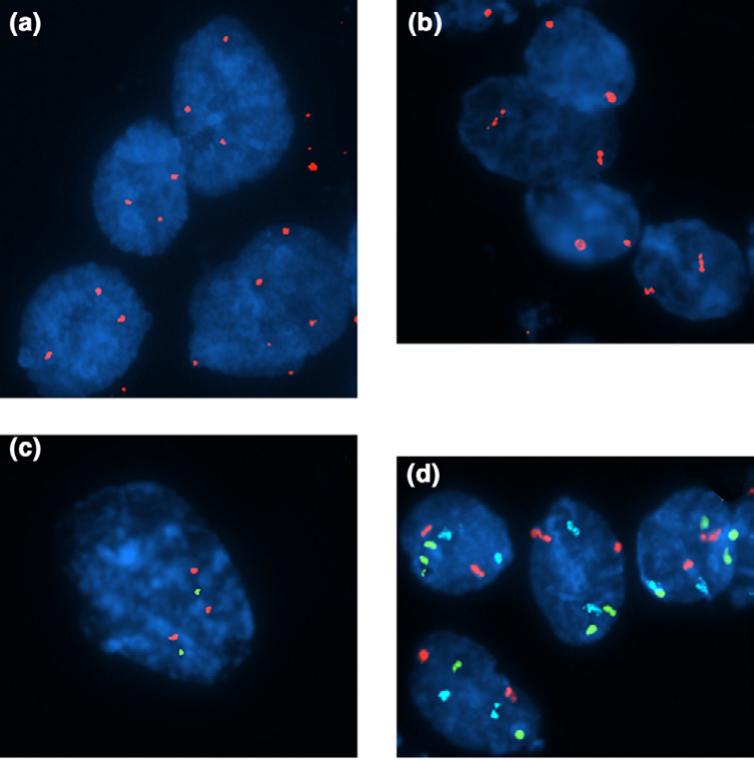
FISH analysis of breast cancer cells from patient B8. **(a) **Analysis with a specific *LRIG1 *(red) probe showing increased gene copy number (more than two copies) of the *LRIG1 *gene at 3p14. **(b) **Analysis with a specific CEP3 (centromeric chromosome 3; red) probe, showing no additional chromosome 3. **(c) **Analysis with a specific 3p subtelomeric probe (green) and *LRIG1 *(red) mixture showing increased gene copy number of the *LRIG1 *gene but only two copies of the 3p arm. **(d) **Analysis with a mixture of probes for CEP3 (red), X chromosome (green) and chromosome 18 (blue), showing no aneuploidy for these chromosomes.

FISH analysis revealed increased copy numbers of *LRIG1 *in four of the nine tumours from group B (example shown in Fig. 1a). The fraction of tumour cells with increased copy number varied between 21% and 49%. Normal signal pattern corresponding to two *LRIG1 *copies per nucleus was detected in the remaining five tumours (Table 2). A parallel FISH analysis including 10 tumours of a different tissue origin showed no aberrations of *LRIG1 *gene copy numbers in these tumours (ongoing study, data not shown). In one of the breast tumours with increased copy number of *LRIG1 *(patient B8), a more detailed FISH analysis was performed to assess the chromosome 3 status and the ploidity of the tumour cells. This showed that the *LRIG1 *copy number was increased (Fig. 1a) but the chromosome 3 centromere was not (Fig. 1b). Furthermore, by using a mixture of *LRIG1 *probe and a specific 3p subtelomere probe (probe position 30 tel (D3S4559); Abbot Vysis), no increased copy number was found for the 3p subtelomeric region either (Fig. 1c). No evidence of aneuploidy was found, as analysed by using centromere probes for chromosomes 3, 18 and X (Fig. 1d).

FISH analysis was also performed on the breast cancer cell lines MDA-MB-231, MDA-MB-415, HS 578T and ZR-75-1, and the immortalised mammary epithelial cell line hTERT-HME1. Increased copy number of *LRIG1 *was found in three of the five cell lines (MDA-MB-231, HS 578T and hTERT-HME1), whereas decreased copy number was found in the MDA-MB-415 cell line. A normal FISH signal pattern for *LRIG1 *was present in ZR-75-1.

### Quantitative RT-PCR

Quantitative RT-PCR was performed on RNA extracted from tumour tissue and non-neoplastic tissue from seven of the nine patients in group B (the quality of the samples from two of the patients was not adequate for quantitative RT-PCR analysis). A fibroadenosis was also examined, with collected pieces both from the fibroadenosis and the surrounding tissue. The expression levels in different parts of the healthy breast tissue from the same individual did not differ by more than 20% (data not shown) and, therefore, a 20% cut-off level was used for both overexpression and underexpression. The ratio between the expression in the tumour and non-neoplastic tissue was calculated and ratios >1.2 were regarded as significant tumour overexpression and ratios <0.8 were regarded as significant tumour underexpression. *LRIG1 *mRNA was significantly overexpressed in two of the seven tumours and significantly underexpressed in two of the seven tumours (Table 3). The three tumours with increased *LRIG1 *copy number (FISH analysis) that were able to be analysed by RT-PCR for LRIG1 showed significant overexpression of *LRIG1 *mRNA in two cases. Four tumours with increased *LRIG1 *copy number were analysed by RT-PCR for EGFR/ERBB1, and all four showed significantly lower expression of EGFR/ERBB1 and three showed significantly higher expression of ERBB2 than their matched normal controls (Table 4). Two of the tumours without increased *LRIG1 *copy number also had lowered EGFR expression.

### Western blot analysis of fresh tumour samples and their matched non-neoplastic breast tissue

**Figure 2:**
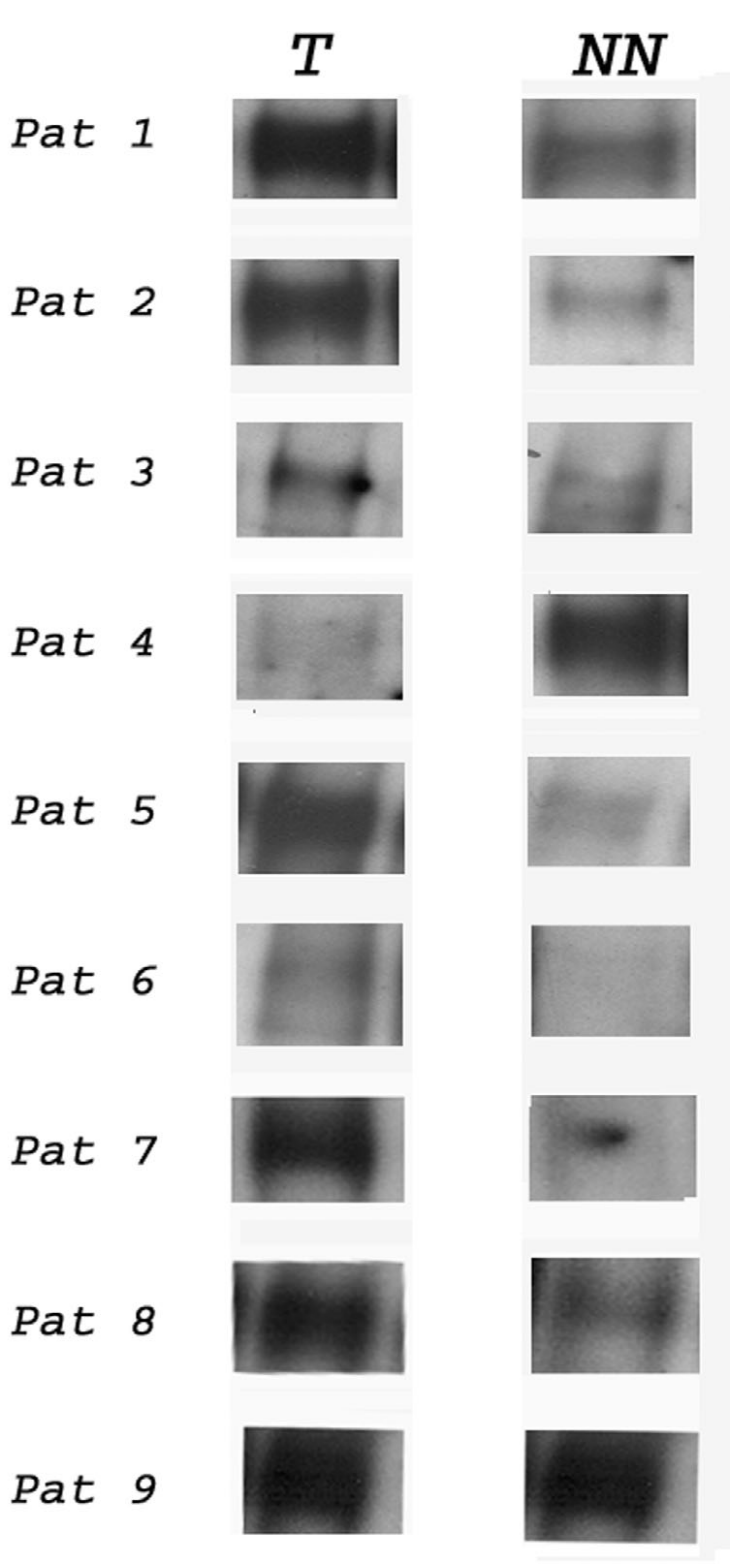
Western blot analyses of LRIG1 in the nine breast cancer patients in group B. Tumours (T) versus non-neoplastic breast tissue (NN). Western blot analysis was performed on samples with primary antibodies LRIG1-151 and anti-rabbit anti-actin. A visual change of at least 50% was considered convincing, as determined by three different investigators. Pat, patient number.

In group B, five of the nine tumours overexpressed the LRIG1 protein compared to their matched non-neoplastic tissues as analysed by western blotting. Four of these five tumours also displayed increased *LRIG1 *copy number (Fig. 2, Table 3). Thus, all of the tumours with increased *LRIG1 *copy number overexpressed LRIG1 as determined by western blot analysis, but also one tumour with normal *LRIG1 *copy number showed high levels of the protein by western blotting.

### Combined analysis of group A and B

In total, 28 breast tumours were analysed by FISH with *LRIG1 *specific probe. A normal signal pattern, corresponding to two *LRIG1 *copies per nucleus, was detected in 16 cases. In 11 out of 28 tumours (39%), an increased number of *LRIG1 *signals were found (Table 2, Fig. 1a). The fraction of tumour cells with increased copy number varied between 21% and 79%. Complementary FISH analyses showed that there was no increase in the copy number of the entire 3p arm (Fig. 1b–d). As seen by quantitative RT-PCR analysis, two out the three analysed tumours with increased *LRIG1 *copy number (in group B) showed higher expression of *LRIG1 *mRNA than the matched non-neoplastic breast tissues. In all four tumours with increased *LRIG1 *copy number, expression of LRIG1 protein was higher than in the matched non-neoplastic breast tissue, as assessed by western blot analysis.

Complementary analysis of five transformed breast cancer cell lines showed similar results, with three of them showing an increased copy number of the 3p14 locus.

## Discussion

This novel investigation of 3p14 demonstrated unexpectedly an increased copy number of the proposed tumour suppressor gene *LRIG1 *in 39% (11/28) of the breast tumours and in 60% (3/5) of the breast cancer cell lines. The malignant process is believed to be driven by genetic diversification through mutations, deletions and amplifications followed by natural selection of surviving and proliferating cancer cells. One result of this process is the enrichment of amplicons harbouring tumour promoting genetic elements, that is, cancer associated genes. Accordingly, the presented results imply that a common, but hitherto unrecognised, breast cancer related gene was located within an amplicon that included the *LRIG1 *locus at 3p14.

Despite numerous genetic studies, increased copy number at the 3p14 locus has, to our knowledge, never previously been reported in primary human breast tumours. Interestingly, however, amplifications at 3p14 have recently been reported in breast cancer-derived cell lines [29,30]. Previous comparative genomic hybridisation (CGH) studies of 3p have generally shown losses and only rarely gains [31]. There are at least four possible explanations why the herein demonstrated increased copy number at 3p14 has not previously been described. First, the area of increased copy number could be relatively small, and so escaped detection by conventional analyses. Second, most studies of this chromosomal area have analysed LOH, and thus have not addressed possible gene amplifications. Third, results obtained by conventional CGH, a method frequently used to detect both gains and losses, are usually difficult to interpret in chromosomal regions close to the centromere, such as the *LRIG1 *locus at 3p14. By employing an alternative CGH methodology using cDNA arrays, an amplicon at 3p14 was described in breast cancer-derived cell lines [29]. Whether this region is co-duplicated with *LRIG1 *at 3p14 has not been addressed but will be the subject of future studies. Finally, a further limitation of conventional and array based CGH methodologies is that they evaluate the mean gene copy number in the analysed sample. FISH, in contrast, has single cell resolution and, thus, is more sensitive and able to detect modest gene copy number changes that could involve only a minority of the tumour cells.

An important question regarding the herein discovered amplicon is its size and the identity of the underlying possible breast cancer gene(s). We have shown by FISH that the area of increased copy number contained *LRIG1 *at 3p14 but was lacking the centromere and the subtelomeric region of chromosome 3. In addition, as discussed above, chromosome 3 has previously been extensively studied by CGH, which rules out the possibility of a common amplicon spanning centromere-distal regions of 3p. From this, we estimate that the putative breast cancer gene is located on chromosome 3, somewhere between the centromere and 3p21. Obviously, the only gene directly demonstrated so far to be duplicated in the analysed breast tumours was *LRIG1*. This raises the question of whether *LRIG1 *itself is a breast cancer gene. LRIG1 has been proposed to interact with and counteract the effects of growth factor receptors such as EGFR/ERBB1 [23,32], thereby functioning as a tumour suppressor. This hypothesis was recently confirmed by molecular studies showing that LRIG1 downregulates ERBB1-4 by enhancing receptor degradation [25,26]. Because EGFR/ERBB1 and ERBB2 are important and frequently overexpressed breast cancer genes, it is unlikely that LRIG1, as an ERBB antagonist, is a tumour promoter. Of course, we cannot exclude that LRIG1 might have other functions, which for tumour promotion could dominate over its ERBB-antagonising effects. According to a recent estimate [33], however, there are 80 genes in addition to *LRIG1 *in the region between the centromere and 3p21 (coordinates 64M-92M), the gene copy numbers of which could potentially have been increased in conjuction with *LRIG1*. These genes encode a variety of different kinds of proteins, of known and unknown functions, including a tyrosine kinase receptor (EPHA3), a protein phosphatase regulatory subunit (PPP4R2), an ubiquitin-conjugating enzyme (UBE1C), and different transcription factors (e.g. TMF1, FOXP1 and POU1F1). The amplicon described by Hyman *et al*. [29] is confined to a 2.7 mb region (coordinates 72M-75M), which include 13 genes but not *LRIG1 *at 66M. Moreover, an amplicon close to the *LRIG1 *locus with the coordinates 60M-64M was recently described in breast cancer cell line MCF-7 [30]. This amplicon contains about 17 genes. Whether the regions at 72M-75M and 60M-64M are increased in copy number in conjunction with the *LRIG1 *locus at 66M was not addressed in the present study but will be the subject of future studies. Clearly, a more refined mapping of the area of increased copy number and functional studies of candidate genes are needed for defining the hypothesised breast cancer gene(s). We conclude, nevertheless, that a common but hitherto unrecognised breast cancer gene is located at or near the *LRIG1 *locus.

Increased *LRIG1 *copy number as detected by FISH was associated with increased mRNA and protein levels in tumours compared to non-neoplastic breast tissue, as determined by quantitative RT-PCR and western blot analysis (Table 3). A concordance between gene overexpression and enhanced mRNA levels is often, but not always, observed [29].

Because ERBB family members are strongly implicated in the aetiology of breast cancer, and because ERBB proteins and LRIG1 interact at the molecular level, we analysed the expression of EGFR/ERBB1 and ERBB2 mRNA. Intriguingly, EGFR/ERBB1 mRNA was significantly underexpressed in all of the four tumours analysed with increased *LRIG1 *copy number. Of these four tumours, three showed significant overexpression of ERBB2 mRNA. EGFR/ERBB1 is overexpressed in 35% to 60% of breast cancers, which correlates with a negative steroid receptor status, increased ERBB2 and VEGF (Vascular endothelial growth factor) expression [7]. The impact of EGFR/ERBB1 overexpression on clinical outcome has not been completely clarified, but in most studies it is considered to be a negative prognostic factor [34]. The combination of increased *LRIG1 *copy number and protein expression and low EGFR/ERBB1 expression might represent a subtype of breast cancer with its own clinical features. To reveal such a biological subtype, a larger number of tumours must be examined.

## Conclusion

We have described, for the first time, frequent increased copy number at 3p14.3 in breast cancer. This attributes a breast cancer associated gene to 3p14 or surrounding plausibly co-amplified regions. In future studies, it will be important to define the genetic element, that is, the breast cancer gene(s) underlying the observed copy number increase, and to examine a greater number of tumours in order to evaluate its clinical significance.

## Abbreviations

CGH = comparative genomic hybridisation; EGFR = epidermal growth factor receptor; FISH = fluorescence *in situ *hybridisation; LOH = loss of heterozygosity; LRIG1 = leucine-rich and immunoglobulin-like domains 1; PBS = phosphate buffered saline; RT-PCR = reverse transcriptase polymerise chain reaction; SSC = saline sodium citrate.

## Competing interests

The author(s) declare that they have no competing interests.

## Authors' contributions

IL is a PhD student in the project and participated in the FISH analysis and the overall analysis of the results. IL was also responsible for writing the manuscript. TH participated in the collection of new breast cancer samples and investigating whether the samples were representative. MT carried out the quantitative RT-PCR analysis, YJ carried out the western blot analysis, IG was responsible for the FISH analysis, SE participated in contact with patients for the collection of breast cancer tumours, and KG was responsible for the collection of the breast cancer tumours from 1987 to 1995. BM, HH and RH were responsible for the overall design and implementation of the study and also for the overall analysis of the material and they helped to draft the manuscript. All authors read and approved the final manuscript.

## Figures and Tables

**Table 1:** Clinical characteristics of the 28 breast tumours included in the study

	n	% of total	Tumours with increased *LRIG1 *copy number (n = 11)	Tumours without increased *LRIG1 *copy number (n = 17)
Tumour type				
Ductal	21	75	10 (82 %)	11(65%)
Lobular	2	7	1	1
Mixed group	4	14	0	4
Medullar	1	4	0	1
Axillar lymph node				
Negative	17	61	6 (54%)	11 (65%)
Positive	10	36	5 (45%)	5 (29%)
Missing value	1	4	0	1
Estrogen receptor				
Positive	21	75	8 (73%)	13 (76%)
Negative	4	14	2 (9%)	2 (12%)
Missing value	3	11	1	2 (12%)
Progesterone receptor				
Positive	18	64	6 (54%)	12 (70%)
Negative	5	18	3 (27%)	2 (28%)
Missing value	5	18	2 (18%)	3 (18%)
Outcome=deceased	10	36	3 (27%)	7 (41%)

**Table 2:** FISH analysis of the *LRIG1 *locus in 28 breast cancer tumours

FISH 3p14.3	Group A (n = 19)	Group B (n = 9)
Increased copy number	7 (36 %)	4 (44%)
Normal copy number	11 (57%)	5 (55%)
Decreased copy number	1 (5%)	0

Group A: tumours collected 1986 to 1996 retrospectively. Group B: tumours collected 2002 to 2003 prospectively.

**Table 3:** Summary of FISH, western blot and RT-PCR analysis of LRIG1 in nine breast cancer tumours

Patient no.	FISH^a^	Western blot^b^	LRIG1 RT-PCR (T/NN)^c^
B1	I	In	1.45^d^
B2^e^	I	In	-
B3^e^	NL	E	-
B4	NL	D	0.85
B5	I	In	1.63^d^
B6	NL	E	0.50^d^
B7	NL	In	0.36^d^
B8	I	In	1.13
B9	NL	E	1.17

^a^FISH: I, increased *LRIG1 *copy number; NL, normal *LRIG1 *copy number. ^b^Western blot: comparison of the staining in tumour tissue (T) and non-neoplastic tissue (NN). A visual increase (In)/decrease (D) of at least 50% was considered convincing. E, equal levels of protein. ^c^LRIG1 RT-PCR T/NN; LRIG1 mRNA expression level in neoplastic (T) tissue samples divided by levels in matched non-neoplastic (NN) tissue samples. ^d^As described in the results, at p.9, ratios >1.2 are regarded as significant overexpression and ratios <0. 8 are regarded as significant underexpression in neoplastic tissue compared to non-neoplastic tissue. Samples were consecutively collected. ^e^Two tumours did not yield RNA of sufficient quality for the RT-PCR analysis.

**Table 4:** EGFR and ERBB2 quantitative RT-PCR mRNA results in eight patients and matched controls (group B)

Patient number	T	NN	T/NN^a^
1^b^			
EGFR	4,677	12,493	0.37
ERBB2	24,066	21,817	1.1
2^b^			
EGFR	3,321	14,519	0.22
ERBB2	61,389	6,666	9.2
4			
EGFR	20,778	14,842	1.39
ERBB2	9,143	6,742	1.35
5^b^			
EGFR	9,730	15,867	0.61
ERBB2	13,506	3,056	4.41
6			
EGFR	1,305	15,871	0.08
ERBB2	10,677	10,080	1.06
7			
EGFR	1,024	12,162	0.08
ERBB2	7,639	5,871	1.30
8^b^			
EGFR	4,936	28,111	0.17
ERBB2	17,106	9,263	1.85
9			
EGFR	5,540	1,437	3.85
ERBB2	7,330	5,540	1.32

^a^T/NN: mRNA expression levels of *LRIG1 *in tumour tissue (T) samples divided by values in matched non-neoplastic (NN) tissue samples. As described in the results, at p.9, ratios >1. 2 are regarded as significant overexpression and ratios <0. 8 are regarded as significant underexpression in neoplastic tissue compared to non-neoplastic tissue. ^b^Patients with increased *LRIG1 *copy number. No RNA from patient 3 was available for EGFR/ERBB2 analysis.
